# Use of the CHM13-T2T genome improves metagenomic analysis by minimizing host DNA contamination

**DOI:** 10.1128/msystems.00840-25

**Published:** 2025-09-10

**Authors:** Donglai Liu, Jinjun Hu, Dan Zhang, Shanshan Ren, Lanqing Zhao, Hongyan Gao, Songnian Hu, Sihong Xu, Guanxiang Liang

**Affiliations:** 1 National Institutes for Food and Drug Control 12540, Beijing, China; 2 Center for Infection Biology, School of Basic Medical Sciences, Tsinghua University 12442, Beijing, China; 3 China National Microbiology Data Center, Institute of Microbiology, Chinese Academy of Sciences 85387, Beijing, China; 4 Tsinghua-Peking Center for Life Sciences 555132, Beijing, China; 5 SXMU-Tsinghua Collaborative Innovation Center for Frontier Medicine, Shanxi Medical University 74648, Taiyuan, Shanxi, China; NYU Langone Health, New York, New York, USA

**Keywords:** CHM13-T2T, metagenomic analysis, host decontamination, bwa-mem

## Abstract

Human-associated metagenomic data often contain human nucleic acid information, which can affect the accuracy of microbial classification or raise ethical concerns. These reads are typically removed through alignment to the human genome using various metagenomic mapping tools or human reference genomes, followed by filtration before metagenomic analysis. In this study, we conducted a comprehensive analysis to identify the optimal combination of alignment software and human reference genomes using benchmarking data. Our findings show that the combination of bwa-mem and the telomere-to-telomere human genome (CHM13-T2T) is the most effective in removing human reads in simulated data. We also analyzed CHM13-T2T-derived sequences in RefSeq to understand how CHM13-T2T reduces false positive results. Finally, we assessed clinical samples and found that CHM13-T2T effectively reduces host-derived contamination, particularly in low microbial biomass samples. This study provides a thorough overview of the application of CHM13-T2T in metagenomic analysis and highlights its significance in improving microbial classification accuracy.

**IMPORTANCE:**

Human gene sequences account for a large proportion of metagenomic sequences. To gain accurate and precise microbiome information, effective host-derived contamination removal methods are required. Both the alignment algorithm and the reference genome could influence the effectiveness of this process. The telomere-to-telomere human genome (CHM13-T2T) is a state-of-the-art human genome with 216 Mbp of additional new sequences compared with the commonly used GRCh38.p14. Our findings show the optimal dehosting effect of CHM13-T2T combined with the bwa-mem software in metagenomic analysis. We also investigate the reasons for the superiority of CHM13-T2T. Our study provides insights into optimal strategies for host sequence removal from metagenomic data. A standard reference is proposed for future metagenomic analysis, which can improve the accuracy of microbial identification.

## INTRODUCTION

Metagenomic next-generation sequencing (mNGS) technology enables rapid and unbiased microbial diagnosis independent of cultivation, and it has been increasingly utilized in clinical microbiology, for diagnosing infections (1). However, metagenomic data from clinical samples often contain unavoidable contamination from human sequences. These sequences can constitute as much as approximately 95% to 99.9% of the mNGS data, especially in low-biomass samples (2, 3). Contamination from host sequences can compromise microbial analysis, potentially leading to incorrect conclusions, while also limiting the utilization and sharing of microbial data due to ethical concerns (4). Furthermore, accurate and comprehensive removal of host sequences is crucial for improving the computational speed and accuracy of downstream analysis (5).

The most commonly employed approach for eliminating host sequences entails discarding reads which are aligned with the human reference genome (6, 7). Both the alignment algorithm and the reference genome could influence the effectiveness of this process. The accuracy of alignment tools varies under different read lengths or different characteristics of the reference genomes (8–10). The human reference genome assembly, maintained by the Genome Reference Consortium, has undergone five major updates. While GRCh38.p14 (referred to as hg38) is the most commonly used reference genome in the current mNGS dehosting process (11). Another reference genome used in many studies is the Chinese Han genome (referred to as YH) from the Yanhuang Project. It is a haplotype-resolved genome assembly sourced from an Asian individual and can be more suitable for mNGS data from Chinese Han individuals’ dehosting (12–14). However, there are still large amounts of unknown sequences distributed in these reference genomes. Recently, Nurk et al. completed the assembly of a complete, telomere-to-telomere human genome (referred to as CHM13-T2T) in 2022, adding approximately 8% (216 Mbp) new sequences, closing all remaining gaps, and correcting thousands of structural errors compared with hg38 (15), which enabled us to further facilitate the discrimination of human-origin contaminative sequences.

In this study, we conducted a benchmarking experiment to evaluate the effectiveness of various strategies for removing host sequences in metagenomic analysis. Our findings revealed that utilizing the bwa-mem algorithm based on CHM13-T2T was the most effective strategy for eliminating host reads. Additionally, we uncovered hidden host contamination in RefSeq microbial genomes and clinical metagenomic samples by leveraging the CHM13-T2T unique segments. This study offers a comprehensive overview of CHM13-T2T’s application in metagenomic analysis and underscores its importance in enhancing microbial classification accuracy.

## MATERIALS AND METHODS

### Data set construction

To benchmark the performance of the bioinformatics methods in diverse biological settings, a series of mock samples and clinical samples were sequenced using Illumina Nextseq-500 (ILMN) and MGISEQ-200 (MGI) platforms, respectively. The mock samples were prepared by mixing 10 different microorganisms into human HeLa cell lines and diluting them accordingly (Table S1). For mock samples, library construction and sequencing were conducted in parallel by four independent laboratories. The libraries were sequenced to 75 bp single-end reads on ILMN and 50 bp single-end reads on MGI. The raw reads were preprocessed by using fastp v0.23.2 (16) to remove adapter sequences, low-quality reads, and short reads. One ILMN library was excluded from the study due to insufficient sequencing yield. In addition, HeLa cells contain human papillomavirus sequences that have been removed in the later data analysis to avoid interference.

### The identification and labeling of host-originated reads

Reads that passed all quality controls were subjected to the host reads removal procedure. The combinations of reference genomes (CHM13-T2T, hg38, and YH) and algorithms were applied to each sequence. This included five algorithms: Bowtie2 v2.4.5 (in very fast and very sensitive mode) (17), bwa mem v0.7.17-r1188 (18), STAT v1.0.2021_05_05 (HRRT, human read removal tool, also known as the sra-human-scrubber) (19), and Kraken2 v2.1.1 (20). In one instance, host read removal using STAT was exclusively conducted with the hg38 genome. This was due to the HRRT distribution containing solely a meticulously crafted database tailored to hg38, rendering replication unfeasible with the other two genomes. In total, 13 strategies were developed. All software was run with default or preset parameters.

Each read was labeled by each alignment strategy to indicate whether it originated from the host. For Kraken2, a TaxID assignment to any Chordata species was considered as the host label. We compared all assigned labels with each read to delineate a judgment standard (namely, gold standard) by imposing the following criteria. A read was assigned a consensus label if concordant results were found for all strategies. If the results were discordant, the read was subjected to further examination. If a read was labeled as host-derived by both an alignment-based method and a k-mer-based method, we designated the host label to that read as the gold standard. The remaining reads were assigned discordant labels and were queried against the NCBI nr/nt database with BLAST (21). If a hit to Chordata sequences was found with sufficiently high alignment quality (identity >90%, coverage >90%), the read was considered truly of host origin; otherwise, it was deemed non-host. Therefore, our gold standard inevitably has a bias toward alignment-based methods. Additionally, because of the partial sequence similarity in the human and bacterial reference genomes, it is inevitable that part of the bacterial reads will be annotated as host reads in our alignment process. However, this is only a small proportion (about 0.1%) and does not affect the abundance of the microbiota in the whole metagenomic analysis. We also attempted a direct labeling strategy, sequencing HeLa and microbes separately, labeling the reads accordingly, and then combining them for downstream benchmarking. We tested the efficiency of our methods using the direct labeling strategy and found the same result as our “gold standard.”

### The evaluation for host removal performance

For each method, the label assigned to each read was compared against the gold standard, resulting in classification of reads as true positive (TP), false positive (FP), true negative (TN), and false negative (FN), where “positive” denotes when a read is labeled as human, and vice versa. The sensitivity, specificity, and Matthew’s correlation coefficient (MCC) were then calculated for all strategies, as defined below (22):


(1) $$sensitivity=\frac{TP}{TP+FN},specificity=\frac{TN}{TN+FP},$$

(2) $$MCC=\frac{TP\times TN-FP\times FN}{\sqrt{\left(TP+FP)(TP+FN)(TN+FP)(TN+FN\right)}}.$$

Sensitivity and specificity, which were widely used and well-interpreted, were used as the evaluation metrics.

For reference, genome-specific FN sequences, custom scripts, and jellyfish v2.3.0 were utilized to compute the guanine-cytosine (GC) content, k-mer frequency, and alignment positions to the CHM13-T2T genome, followed by extraction and visualization (23). The taxonomic classification of all TN and FN host sequences was performed using Kraken2 v2.1.1, with the NCBI RefSeq databases for archaea, bacteria, fungi, viruses, and protozoa as references (20, 24). Principal component analysis (PCA) of the microbial species composition in clinical samples was conducted using FactoMineR v2.5 and factoextra v1.0.7 (25).

True positive microbial sequences were defined as those whose gold-standard host label was negative, and their microbiological classification by Kraken2 v2.1.1, utilizing the default RefSeq database, corresponded to either spiked-in microbes in mock samples (Table S1) or aligned with the culture or FilmArray results in clinical samples (Table S2). False negative microbe calls were defined as sequences with positive host labels in the gold standard but not removed by a removal method, and their Kraken classification did not belong to Chordata.

### The host contamination assessment of microbial genomes in RefSeq

Initially, Conterminator software was used to make an overall alignment between the microbial genomes in RefSeq with Hg38 and CHM13-T2T assembly distinctly (11, 24, 26). Hg38 was aligned to CHM13-T2T using Winnowmap v2.0.3 to extract the exclusive regions of CHM13-T2T (27). Then Blastn was applied to align the exclusive sequences to microbial taxa in RefSeq (Protozoa, Fungi, Archaea, Bacteria). Exclusive sequences of CHM13-T2T exhibiting an alignment length of at least 100 bp with an identity of over 90% were segmented into non-overlapping 100 bp k-mers. Sequences matching two k-mers with target microbial genomes (two species in the bacterial database) were identified as potential contaminants. To quantify the extent of contamination, the ratio of the length of contaminant sequences to the total length of the respective microorganism genomes was calculated. Genomes with a contamination ratio >0.01% were labeled as severely contaminated.

### Application of CHM13-T2T in the metagenomic de-hosting process of clinical samples

At first, metagenomic data of newborn (month0), one-month (month1), and 4-month-old (month4) infants were downloaded from Liang et al. (28). After quality control, the host removal process was performed by bwa-mem with default parameters, using hg38 as the human reference genome. The resulting sequences were aligned to the CHM13-T2T exclusive sequences using bwa-mem. The aligned sequences were sorted with the standard library in Kraken2. Then, organize and graph the classification results using pavian (29).

### Statistical analysis

To compare the sensitivity, specificity, and MCC of all methods, the normality of the distribution of their difference was tested using the Shapiro-Wilk test. Non-parametric test was used for comparison. The Kruskal-Wallis test was used as the omnibus test, and the Wilcoxon signed-rank test (one-sided) with false discovery rate adjustment was used as a post hoc test for pairwise comparison. When comparing the performance of algorithms, the reference genome used was held invariant to determine which algorithm served the best for a particular genome, while when comparing the performance of the reference genomes, the best-performing genome-algorithm combination was used to exclude the effect of the algorithms. Finally, to further investigate the unmappable host reads (FN reads) to each genome, we conducted analysis of variance and unpaired two-sided *t*-tests to compare the GC contents of the unmapped reads. All statistical analyses were performed using R (v4.1.2), and statistical significance was defined as a *P* value <0.05 in all cases.

## RESULTS

### The bwa-mem algorithm based on CHM13-T2T performs best in host decontamination

**Fig 1:**
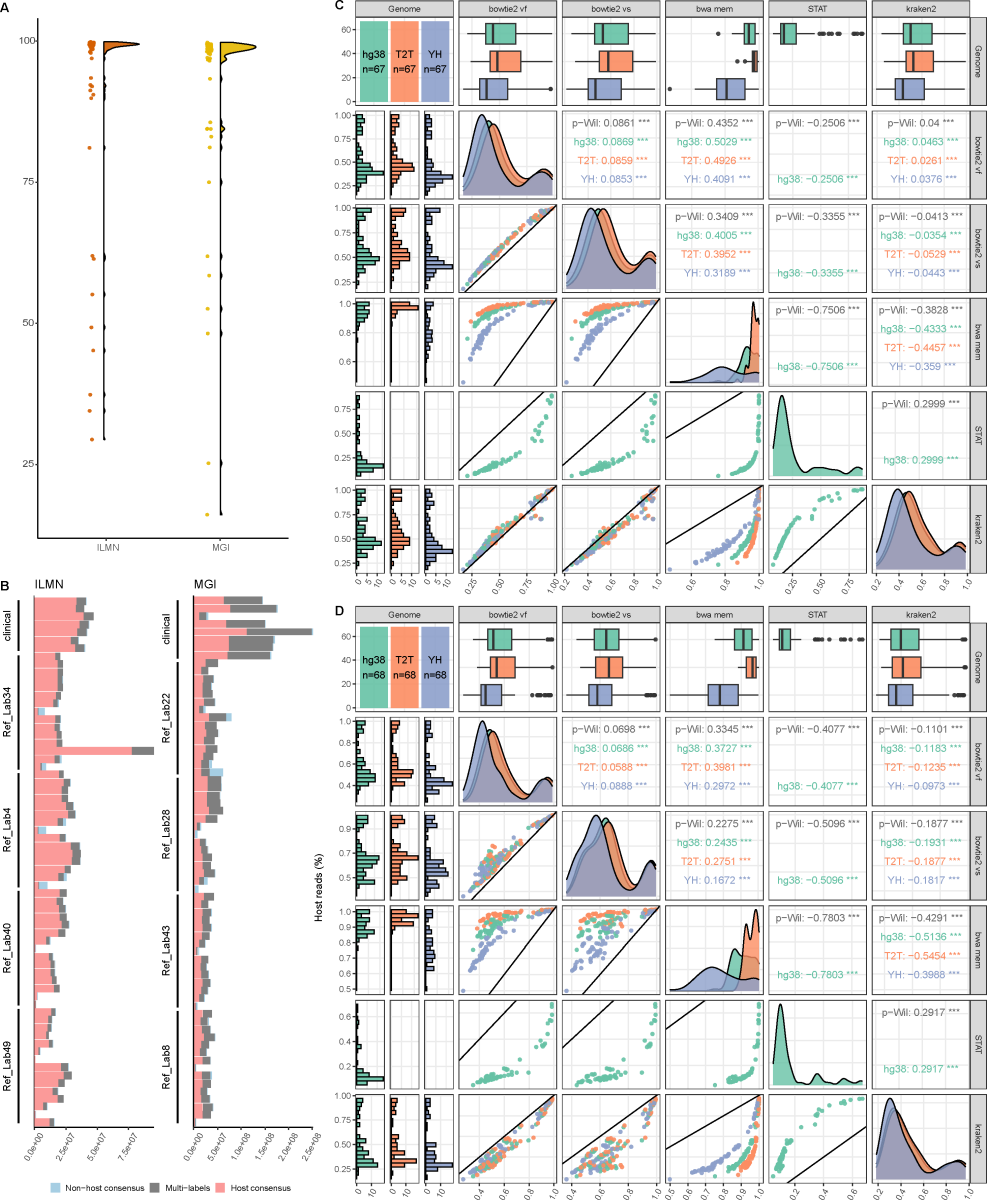
Summary of the performance comparisons. (A) Distribution of host reads percentage of all samples, for ILMN (red) and MGI (yellow). (B) The total number of reads in each sample. Reads are grouped based on the labels given by all host removal methods: non-host consensus (blue), host consensus (red), and multi-labels (gray). (C and D**)** MCC comparison across all reference genomes and relevant algorithms. The first column represents the MCC of each sample calculated with different algorithms versus different genomes: hg38 (green), CHM13-T2T (orange, abbreviated as “T2T”), and YH (purple). The first row represents the sum of MCC values for each calculation. The diagonal represents the density distribution of the boxplot. Plots in the upper triangle: paired Wilcoxon (p-Wil) comparisons between each combination of reference genomes and algorithms. Numbers are the median of differences between the pair, asterisks show the significance of adjusted *P* values (no label: insignificant, *: <0.05, **: <0.01, ***: <0.001), and numbers in colors show the comparison for each genome. Plots in the lower triangle: scatterplot of MCC. The black line has a slope of 1 and an intercept of 0; therefore, points (each representing a sample) above it have a higher MCC value on the *y*-axis than on the *x*-axis and vice versa. Abbreviations: vf, very fast; vs, very sensitive. Results of different sequencers were arranged for (C) ILMN and (D) MGI.

We utilized a combination of five alignment algorithms (bowtie2 vf, bowtie2 vs, bwa mem, STAT, and Kraken2) and three reference genomes (CHM13-T2T, hg38, and YH), resulting in 13 different strategies that were compared against the mNGS data set from this study to determine the optimal host decontamination method. Fifteen simulation metagenomic samples and eight clinical samples from two different sequencing platforms (ILMN and MGI) were used to test the effectiveness of 13 strategies. About 80% of the samples from the standard library exhibited a host content exceeding 90%, while some highly diluted samples (such as D1-1[100], D2-1[100]) exhibited lower host content (Table S3 and S4; Fig. 1A). This may be due to the potential introduction of contaminating microbes during sample dilution. We then assessed the robustness of our pipeline. The “high-confidence host reads” identification scheme was defined in order to effectively evaluate the merits of various host decontamination methods (Fig. S1). Approximately 55.6% of sequences across all data samples were consistently labeled as host sequences across all 13 strategies (Fig. 1B).

All reads were categorized as true positive (TP), false positive (FP), true negative (TN), and false negative (FN), by comparing the labels of each method to the gold standard. MCC, sensitivity, and specificity per read of all methods were calculated and compared pairwise (Fig. 1C and D; Fig. S2 and S3). Further analysis revealed that the performance of the bwa-mem algorithm was significantly superior to other alignment algorithms (Fig. 1C and D; Fig. S2 and S3). The median differences in MCC and sensitivity for host decontamination results relying on the bwa-mem alignment algorithm were both greater than 0.34 and 0.23, compared with other methods (Kruskal-Wallis test, *P* < 0.001; Fig. 1C and D; Fig. S2). Following these were bowtie2-vs, Kraken2, and bowtie2-vf algorithms (Fig. 1C and D; Fig. S2). It is worth noting that STAT was the most specific algorithm, although its sensitivity and MCC were lower than other algorithms, suggesting that the removal criteria of STAT were relatively more conservative (Fig. S2 and S3).

More importantly, employing CHM13-T2T as the reference genome consistently improved the sensitivity of host sequence classification and overall performance across all algorithms (Fig. 1C and D; Fig. S2 and S3). Compared with hg38, CHM13-T2T with the bwa-mem algorithm showed an increase in MCC values by 3.15–4.46% (*P* < 0.001; Table 1) and sensitivity by 0.05–0.14% (*P* < 0.001; Table 1). Compared with YH, the increase of MCC could reach 14.84–16.41% (*P* < 0.001; Table 1), and sensitivity increased by 0.33–0.72% (*P* < 0.001; Table 1).

**TABLE 1:** Improvement of performance when switching to CHM13-T2T

Sequence	Index	CHM13-T2T compared with hg38		CHM13-T2T compared with YH	
		Median of differences (pp.)^*a*^	Adjusted *P* value	Median of differences (pp.)	Adjusted *P* value
ILMN	MCC	3.1453	1.15E−12	14.838	1.15E−12
	Sensitivity	0.0534	1.15E−12	0.3269	1.15E−12
	Specificity	−0.0063	1	−0.006	1
MGI	MCC	4.4551	7.82E−13	16.4127	7.82E−13
	Sensitivity	0.1443	7.82E−13	0.7238	7.82E−13
	Specificity	0.0005	1	0.0006	1

^*a*^ pp.: percentage points.

### Employing CHM13-T2T reduces the FN and FP calls in microbial detection

**Fig 2:**
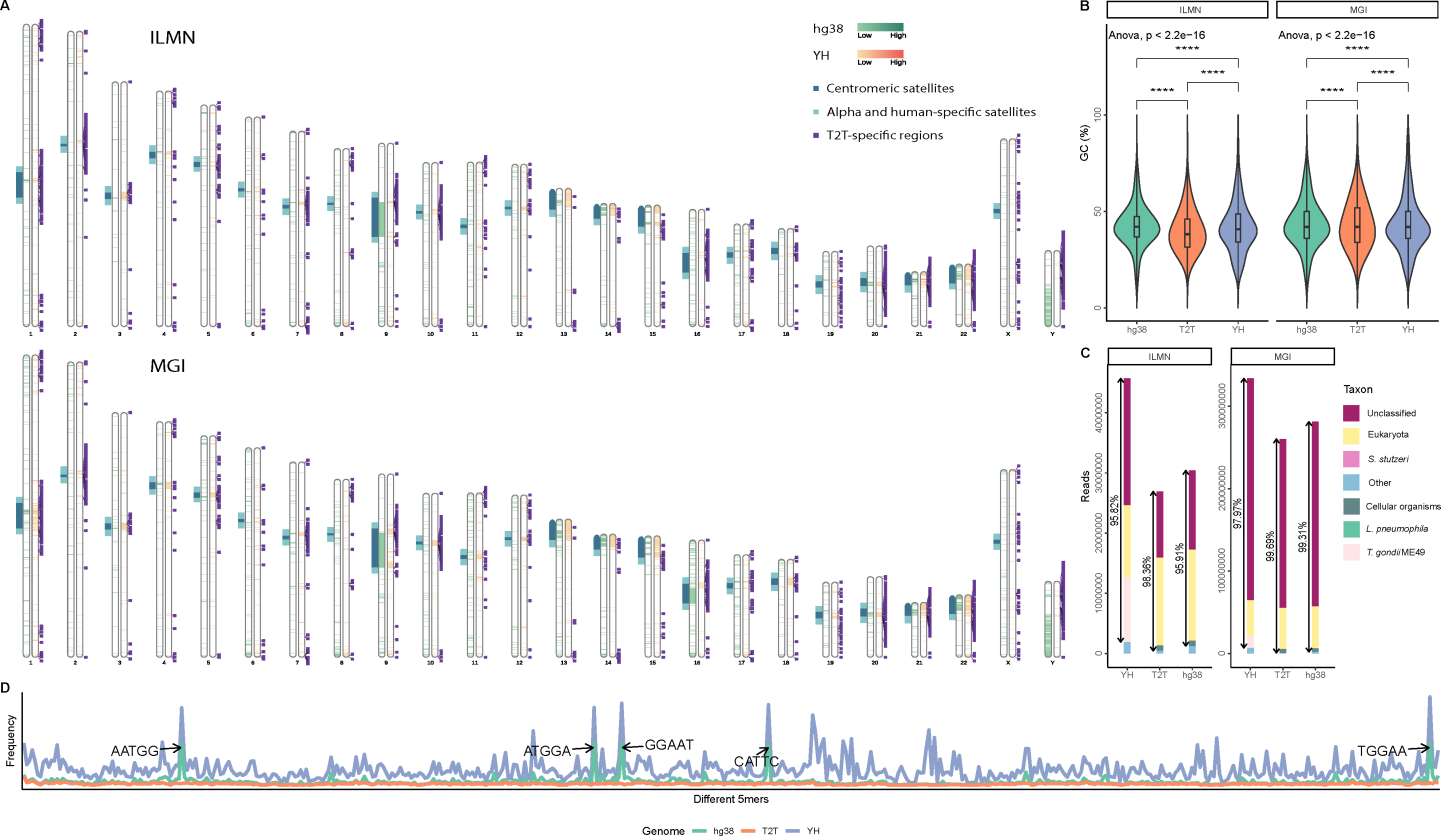
Characteristics of the uniquely mappable reads. (A) Ideogram of the CHM13-T2T assembly, showing the genomic location of reads unmappable to the other reference genomes (for each chromosome, left column in green: hg38, right column in orange: YH); regions newly added therefore unique to the CHM13-T2T assembly (purple); centromeric (deep blue) and alpha/human-specific (light blue) satellite arrays. (B) Distribution and comparison of the GC content of reads unmappable to each reference genome. (C) Distribution of taxa falsely assigned to true host reads. Only the top five taxa are shown separately, the rest are tallied together. Percentages are the proportion made up by the top 5. (D) The k-mer spectrum analysis of false negative reads for each genome. “CHM13-T2T” was abbreviated as “T2T.”

Subsequently, we queried the effects of using CHM13-T2T compared with the other two reference genomes on the removal of host sequences. We extract the reads mapped to the newly added regions of CHM13 that were misclassified as non-host and conduct a basic functional annotation. The results showed the FN sequences obtained based on hg38 and YH primarily stemmed from the newly added regions of CHM13-T2T. For instance, in telomeres, the short arms of proximal chromosomes and subtelomeric regions, notably in the telomeric regions of chromosomes 1, 2, 3, 4, 5, 7, 9, 10, 16, and 20, as well as the short arms of chromosomes 13, 14, 15, 21, and 22, this distribution pattern aligned with the newly added regions in CHM13-T2T, which are highly repetitive (Fig. 2A). Though most of the low-complexity reads had been removed, the FN sequences from highly repetitive regions of CHM13-T2T were hardly eliminated. Therefore, the low complexity features of these special reads were the main driver of misclassification. On one hand, true host reads that were unmappable to each genome showed a difference in their average GC content (Fig. 2B). However, this difference was inconsistent across the sequencing platforms, and therefore was possibly the effect of sequencer bias, instead of reference genomes. On the other hand, over 87.7% of the 75 bp ILMN FN reads, over 91.4% 50 bp MGI FN reads were assigned by Kraken2 as unclassified, cellular organisms and eukaryote, and CHM13-T2T has the lowest proportion of FN reads classified as bacteria and viruses. (Fig. 2C). Further k-mer analysis revealed a significant presence of tandem repeats of (GGAAT)n originating from the human satellite DNA families II and III (Fig. 2D) (30). Possibly thanks to its revamping of the satellite regions, CHM13-T2T had a much lower number of errors compared with other human reference genomes.

**Fig 3:**
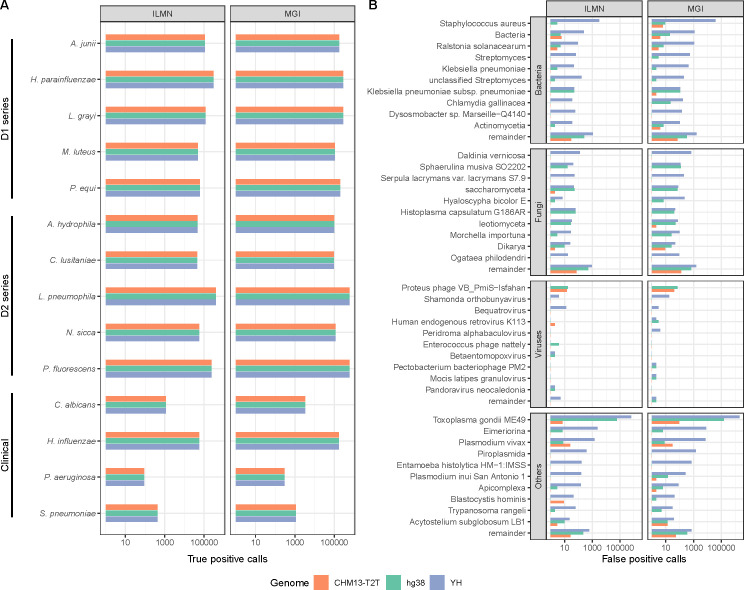
Impact of using CHM13-T2T as reference genome on the results of microbe detection. (A) The true positive microbial sequence classification calls. A comparison between the results of all the reference genomes is shown for both mock (D1 and D2 series) and clinical samples, across three sequencing platforms. (B) The false-positive microbial sequence classification calls. The taxa reported by Kraken2 are hereby categorized into bacteria, fungi, viruses, and others, which mostly include amoebas and apicomplexans. Only the top 10 taxa are shown, and all sequences classified as other taxa are tallied into the remainder group.

Host decontamination strategies using different reference genomes had minimal impact on detecting true microbial positives, as all the known microorganisms were correctly identified in the standard sample data set (Fig. 3A). The corresponding PCA analysis also found no difference between the results of all three genomes (Fig. S4). However, the utilization of CHM13-T2T significantly reduced FP microbial detection (Fig. 3B). Specifically, employing CHM13-T2T resulted in a decrease in FP microbial species detection rates of approximately 47.75% and 75.85% compared with hg38 and YH, with a corresponding reduction in FP microbial sequence counts of approximately 99.60% and 99.97%. It is noteworthy that a significant proportion of FP sequences (~2.8 Mbp, constituting approximately 10.8%) among all host decontamination methods were erroneously identified as *Toxoplasma gondii ME49*, while using CHM13-T2T reduced the detection of these sequences by around 2,148 and 26,403 times compared with hg38 and YH. Additionally, using CHM13-T2T completely eliminated FP detections of *Eimeriorina* and *Piroplasmida*. A study reported *Toxoplasma gondii* reference genome (GCA_000006565.2) in the RefSeq contained contaminated sequence from human sources (31). These results indicated that using CHM13-T2T as the reference genome could achieve more precise filtering of human-derived reads, thereby generating more accurate profiles of microbial communities.

### CHM13-T2T facilitates uncovering more contaminants in microbial genomes

As mentioned above, employing CHM13-T2T reduced the false positive calls in microbial detection. Next, we aimed to investigate why using CHM13-T2T as the reference, compared with hg38, could detect new host contamination. Microbial genomes from the RefSeq database were used as repositories of contamination sequences to be tested. Conterminator was employed to perform a rapid scan of a representative microbial genome database (495 fungi species, 1,405 archaea species, 95 protozoa species, 5,258 bacteria species) to identify potential contamination originating from either hg38 or CHM13-T2T. As a result, more human-derived sequences within all four taxonomic databases were revealed when considering CHM13-T2T as the putative source of contamination (Table S5).

**Fig 4:**
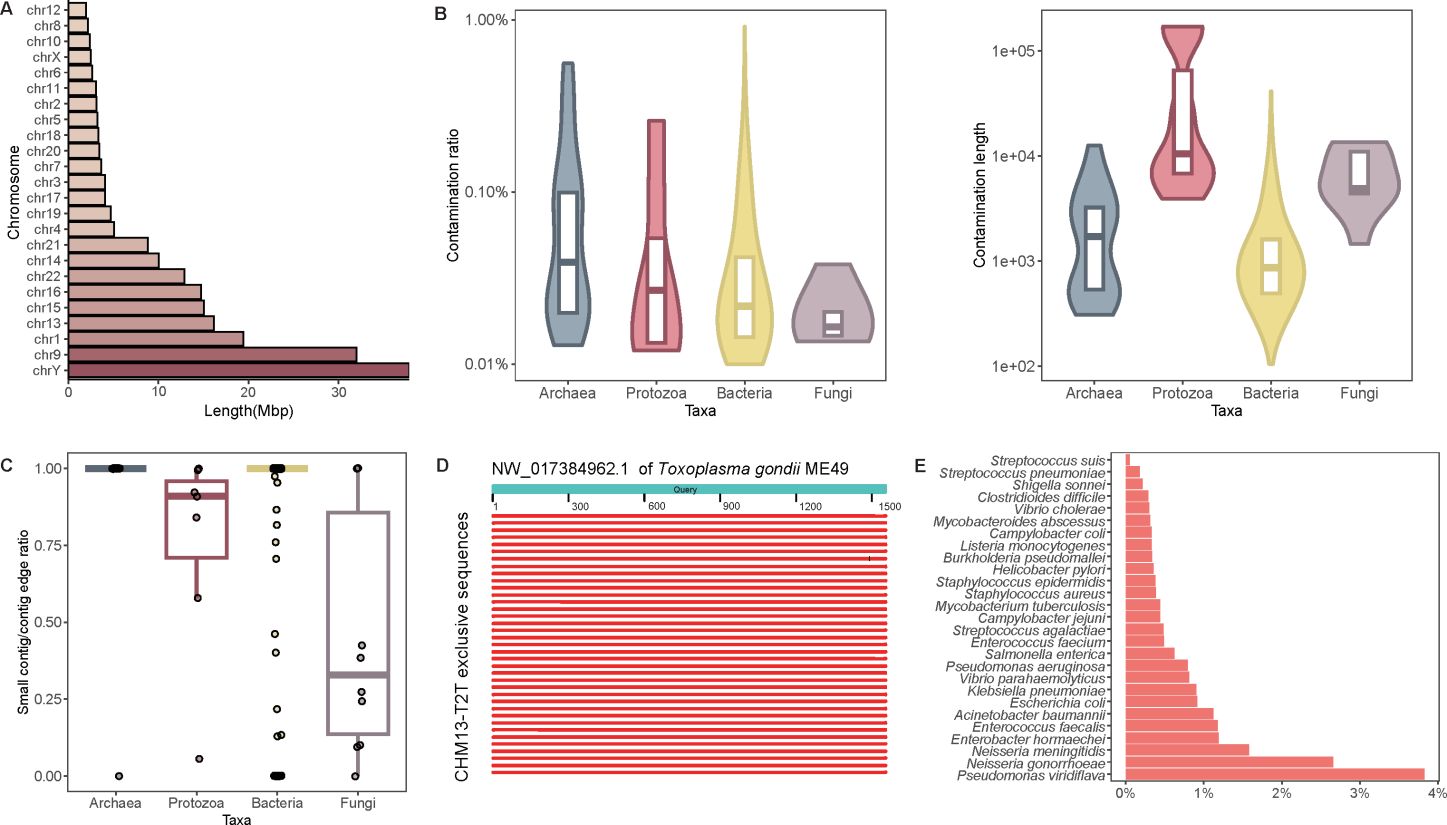
CHM13-T2T contaminants in microbial genomes in RefSeq. (A) Length of the CHM13-T2T exclusive sequences on each chromosome. (B) Distribution of contamination ratio (left) and length (right) in four microbial taxa. Axis *y* was log-transformed. (C) Boxplot of the ratio of contaminant on small contigs or the edge of the contig. Data on viruses was excluded because of their naturally short genomes. (D) Partial alignment diagram between contig NW_017384962.1 of *T. gondii* and CHM13-T2T exclusive sequences. (E) Proportion of genome contamination in over 1,000 bacterial species' genomes compared with the total number of bacterial genomes recorded in RefSeq.

Next, a total of 216 Mbp sequences exclusive to the CHM13-T2T genome were extracted through the alignment between the CHM13-T2T and hg38 for excavating potential microbial contamination within these exclusive regions. Consistent with previous studies, CHM13-T2T exhibited the longest exclusive sequences on chromosome Y, with chromosome 9 containing the second longest gaps (Fig. 4A). The distribution of contaminant sequences on the CHM13-T2T exclusive fragments to draw a landscape of the contaminant patterns in four taxa was summarized (Fig. S5). Chr9: 48424795–76854863 contained the longest contaminant, and 56.01% of length of these intervals contaminated the protozoa database; 26.49% of the fragment Chr16: 41404411–52028806 was the source of contamination in the archaea database. Overall, protozoa genomes suffered the highest level of contamination, with human genetic fragments detected in 55.79% of cases (53 out of 95 genomes). Furthermore, 0.42% of fungal genomes harbored sequences that were contaminated by the CHM13-T2T exclusive sequences. Particularly, the bacterial database encompassed 275,829 genomes, among which 1,912 genomes were found to align with these human-originated sequences.

For each contaminated genome, the contamination ratio was calculated to assess the contamination status of each genome. We focused our attention on severely contaminated genomes with contamination ratios exceeding 0.01% (Table S6). Genomes of archaea had the highest average contamination ratio, while protozoal genomes contained the longest contaminants (Fig. 4B). Furthermore, our investigation revealed that among the highly contaminated genomes, a majority of the contaminated fragments were localized within the small contigs or situated at the edges of larger contigs (Fig. 4C).

As some protozoal and fungal species are parasitic in humans, it is difficult to obtain pure strains *in vitro* from the host environment. This may result in contamination during nucleic acid extraction and sequencing steps. For instance, the genome of *Toxoplasma gondii* ME49 (*T. gondii* ME49) showed a contamination rate of 0.259% associated with the CHM13-T2T insertions. A contig NW_017384962.1, with a total length of 1,557 bp, exhibited alignments with over 90% identity to 1,054 CHM13-T2T insertion fragments (Fig. 4D). Further investigation revealed that these inserted sequences were distributed across the HSat sequences of multiple human chromosomes and up to 233 contigs of *T. gondii* ME49 exhibited similar alignment patterns with the CHM13-T2T insertion sequences. Another example in the fungal taxonomic group is *Malassezia globosa* CBS 7966, a fungus parasitic on the human skin surface known to cause dermatitis. This fungus thrives in oily environments and is challenging to culture *in vitro*. A contig with a length of 897 base pairs, NW_001849834.1, exhibited high identity alignments with 302 regions of the chromosome 9 HSat sequences.

In the realm of bacterial taxa, several “hot” species are attracting research attention continuously. These bacterial species are well-documented in the RefSeq database and often have high-quality genome assemblies due to a high level of attention. However, the results unveiled that some genomes were suffering heavy contamination (Fig. 4E). For example, among the 1,054 *Neisseria gonorrhoeae* genomes examined in this study, 28 were found to contain contaminative sequences from the CHM13-T2T exclusive sequences, along with 26 *Neisseria meningitidis* genomes out of 2,280 that were similarly identified as contaminated from the CHM13-T2T exclusive sequences. Furthermore, our examination extended to another prominent bacterial species, *Acinetobacter baumannii*, which boasts an extensive presence in RefSeq with 7,408 genome entries. Among these, 83 genomes were found to be contaminated, and notably, 52 genomes contained over 0.01% of their total length comprised of the CHM13-T2T exclusive sequences.

### CHM13-T2T utilization enhances the accuracy of microbial classification in clinical samples

**Fig 5:**
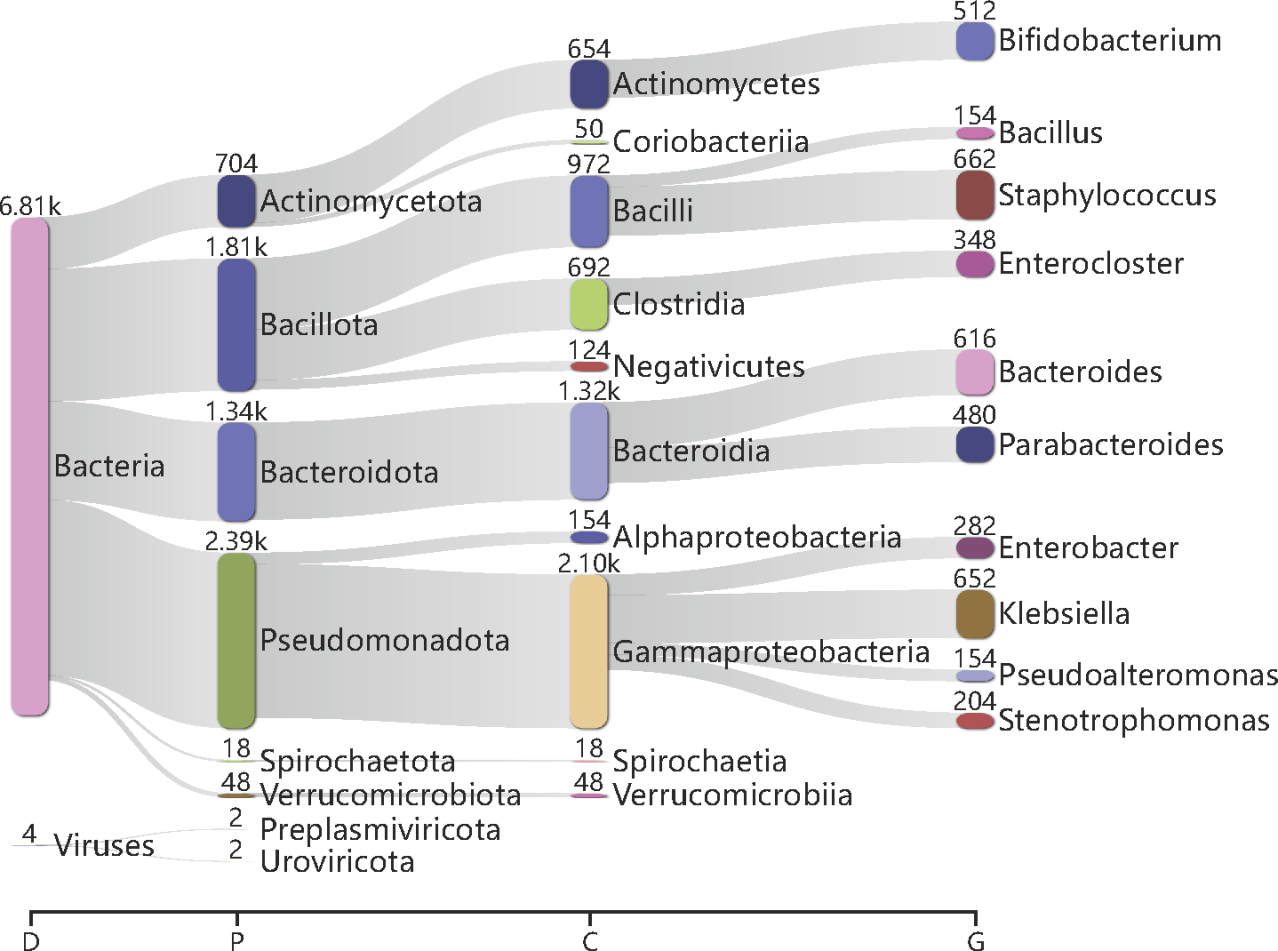
Taxonomy of microbial false positive sequences in clinical samples after removing host reads by hg38. We show the taxonomic ranks domain, phylum, class, and genus. Numbers shown above each taxonomic node indicate the total number of contaminated sequences.

In order to verify the effect of CHM13-T2T during the mNGS dehosting process for clinical samples, metagenomic data of month0, month1, and month4 infants that had been dehosted by hg38 were matched to the CHM13-T2T exclusive sequences using bwa-mem. The results showed that after the routine dehosting process, the human-derived sequences in metagenomic data of month0 infants still accounted for a median of 0.08%, and up to 2.48%, while the human-derived sequences in metagenomic data of month1 and month4 infants were 0%. This may be due to the fact that the majority of the total metagenomic DNA was derived from the host in newborn samples, suggesting that CHM13-T2T will contribute to the host sequence removal in low-biomass microbial samples rather than the high-biomass microbial samples. Taxonomic analysis of human-derived sequences which still retained in the metagenomic data of month0 infants showed nearly half of the reads were correctly classified, and the vast majority were considered to be bacteria, mainly covering *Actinomycetota*, *Bacillota*, *Bacteroidota,* and *Pseudomonadota* phylum (Fig. 5). Among them, *Staphylococcus*, *Bacteroides,* and *Klebsiella* were identified in large numbers. In addition, two viruses, *Preplasmiviricota* and *Uroviricota*, were identified. Therefore, the use of the CHM13-T2T genome in high-host clinical metagenomic data can filter more accurately out human reads and reduce the detection of false-positive microorganisms and viruses, especially *Actinomycetota*, *Bacillota*, *Bacteroidota*, *Pseudomonadota* microorganisms, and *Preplasmiviricota*, *Uroviricota* viruses.

## DISCUSSION

The identification and removal of human sequences is a crucial step in metagenomic analysis. The choice of accurate human reference genomes and efficient alignment algorithms significantly influences the effectiveness of this process. In this study, we evaluated different host removal strategies by combining various human genomes with alignment algorithms and compared their performance on standard mNGS data. We also discovered hidden contamination from host organisms in RefSeq microbial genomes and clinical metagenomic samples by using unique segments from the CHM13-T2T reference genome. To the best of our knowledge, this is the first study to assess the best approach for removing host sequences in metagenomic analysis and to thoroughly examine potential contamination from the CHM13-T2T genome.

Although previous reports have indicated that bwa-mem and Bowtie2 algorithms perform similarly for short read alignment, BWA has been noted to have better performance in detecting human reads (32, 33). The effectiveness of host sequence removal also varied depending on the reference genome used, due to differences in assembly quality. Specifically, CHM13-T2T contains many non-repetitive and coding regions on the short arms of centromeres and proximal chromosomes, contributing to some of these variations (15, 34). In addition, a large number of (GGAAT)n tandem repeats from the human satellite sequence II and III families were not correctly detected by the hg38 and YH genomes (35), suggesting that there were assembly errors in the upstream and downstream intervening sequences, and they could not be aligned with the hg38 and YH genomes. These regions had been greatly improved in the CHM13-T2T genome (34, 36). Therefore, the inclusion of new regions in the CHM13-T2T genome and the enhancements in its assembly quality were key factors in improving host sequence removal. However, during the dehosting process using bwa-mem with CHM13-T2T, some sequences were still incorrectly identified as specific microbial species, leading to false-positive microbial detections. This issue was linked to human-derived contamination present in the RefSeq database (26, 37, 38). Blastn comparison was utilized to verify that among the false-positive detections caused by hg38, high-abundance genomes had regions similar to human reference genomes. It was suggested that human contamination in microbial genomes was one of the causes of false positives. Homologous sequences of microorganisms and human, chimeric sequences of human origin could also cause false-positive detection of microorganisms (39). A high-quality assembled genome could also help avoid false-positive detection due to homology and chimerism.

We also analyzed the CHM13-T2T-derived contamination in microbial genome databases, which highlights the significance of CHM13-T2T in improving our ability to detect and quantify such contamination. This represents a significant expansion of our understanding of the human genome, particularly with respect to previously unknown regions such as chromosome Y (40). The identification of contamination within the microbial genomes from these exclusive sequences has been a concern (41). These previously undetected sequences have now come to light due to the enhanced completeness and accuracy of the CHM13-T2T assembly. The proportion of contaminated genomes varies among different microbial taxa. The calculation of contamination ratios defines the contamination status of genomes, providing a measurement of the contamination in specific organisms. Most human contaminants are present at small contigs due to poor quality of sequencing and assembling results (37). Also, the study finds that highly contaminated genomes often contain contaminated fragments within small contigs or at the edges of larger contigs. This information indicates that some poorly assembled contigs may be due to contamination and can guide future efforts to clean genomic data effectively (42). These conclusions were verified in the subsequent analysis of clinical metagenomic sequences. Therefore, the combination of the bwa-mem algorithm and the CHM13-T2T genome can be used as a better method for the clinical microbial host removal process.

### Conclusion

In conclusion, the selection of the comparison algorithm and reference genome has a significant influence on host removal. The combination of the bwa-mem algorithm and CHM13-T2T can significantly reduce the false positive calls detected by downstream microorganisms, which will contribute to improved microbial genome assemblies. The CHM13-T2T exclusive sequences are a key reason for the stronger host-removal ability of CHM13-T2T. Residual host contaminations were found in both RefSeq and clinical samples aligned with the CHM13-T2T exclusive sequences. In the future, in microbiome research, particularly in metagenomics or pathogen detection, the human CHM13-T2T genome can serve as a reference to identify and remove contaminating segments from microbial genomes and aid in the more effective removal of human-derived contaminants.

## Data Availability

Raw sequence data and sample information can be accessed in the National Genomics Data Center, Institute of Microbiology, Chinese Academy of Sciences (NMDC10019351), publicly accessible at https://www.nmdc.cn.

## SUPPLEMENTAL MATERIAL

Fig. S1 msystems.00840-25-s0001.pdf Workflow of collating host reads removal results of all methods.

Fig. S2 msystems.00840-25-s0002.pdf Comparison of sensitivity.

Fig. S3 msystems.00840-25-s0003.pdf Comparison of specificity.

Fig. S4 msystems.00840-25-s0004.pdf Principal component analysis of commensal organisms in the clinical samples.

Fig. S5 msystems.00840-25-s0005.pdf The contaminative patterns of the T2T exclusive sequences for the database of the 4 taxa.

Legends msystems.00840-25-s0006.docx Legends for supplemental tables and figures.

Supplemental tables msystems.00840-25-s0007.xlsx Tables S1 to S6.

ASM does not own the copyrights to Supplemental Material that may be linked to, or accessed through, an article. The authors have granted ASM a non-exclusive, world-wide license to publish the Supplemental Material files. Please contact the corresponding author directly for reuse.
