# Supplementary figures and images for: Use of the CHM13-T2T genome improves metagenomic analysis by minimizing host DNA contamination
Source: mSystems. 2025 Sep 10;10(10):e00840-25. doi: 10.1128/msystems.00840-25 (PMC12542756; doi:10.1128/msystems.00840-25)

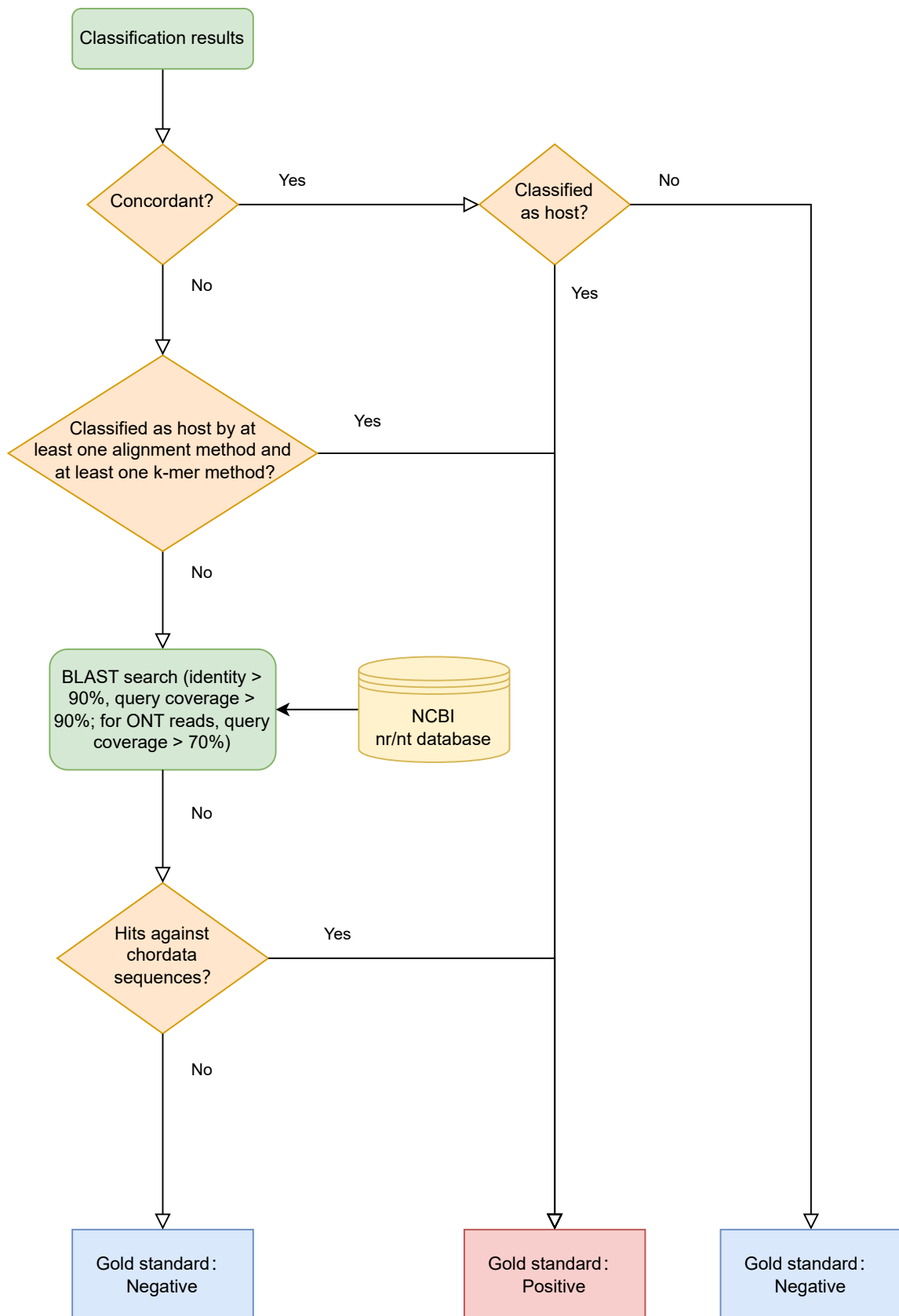

Supplement: Fig. S1 — Workflow of collating host reads removal results of all methods. [file msystems.00840-25-s0001.pdf]

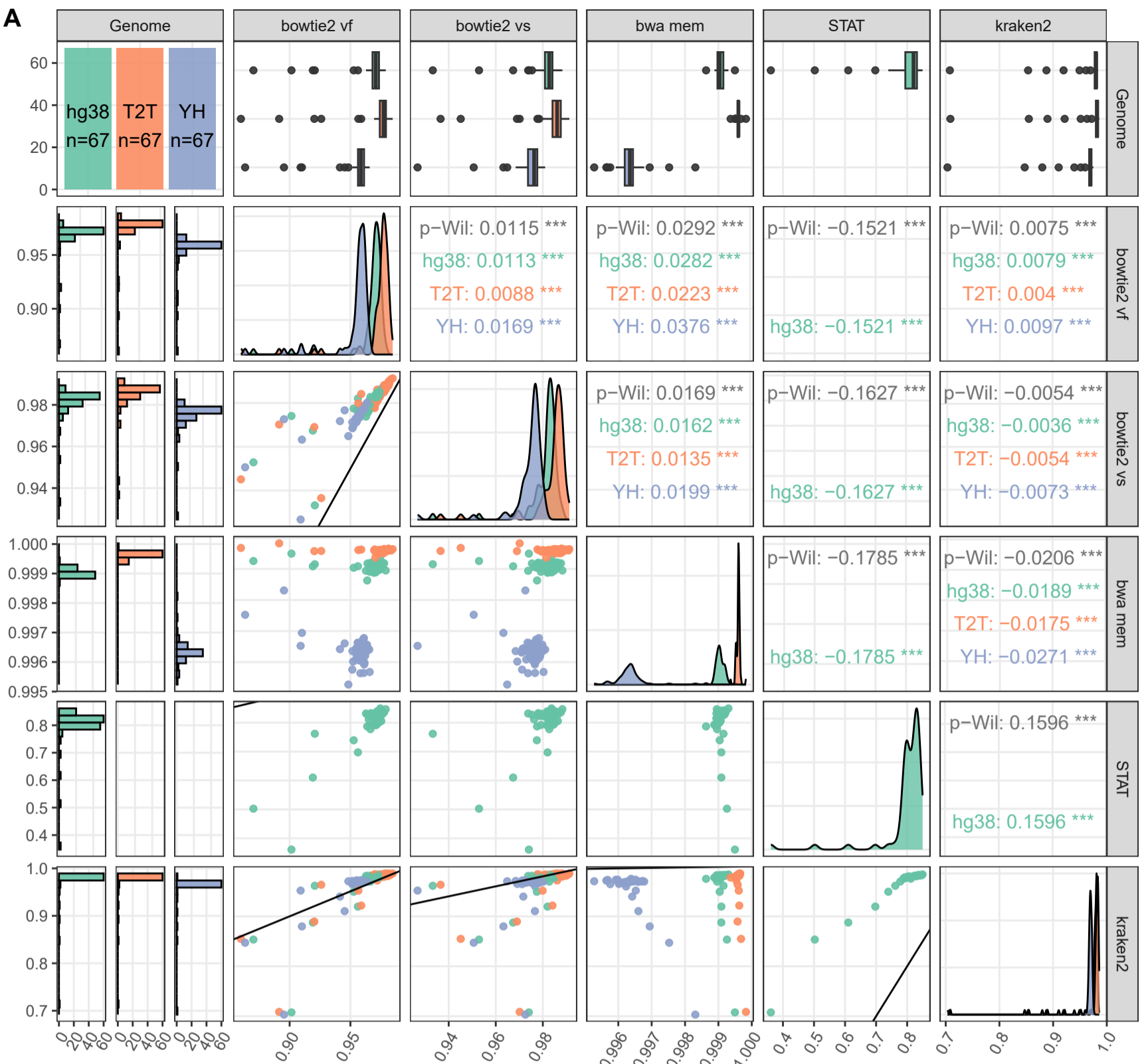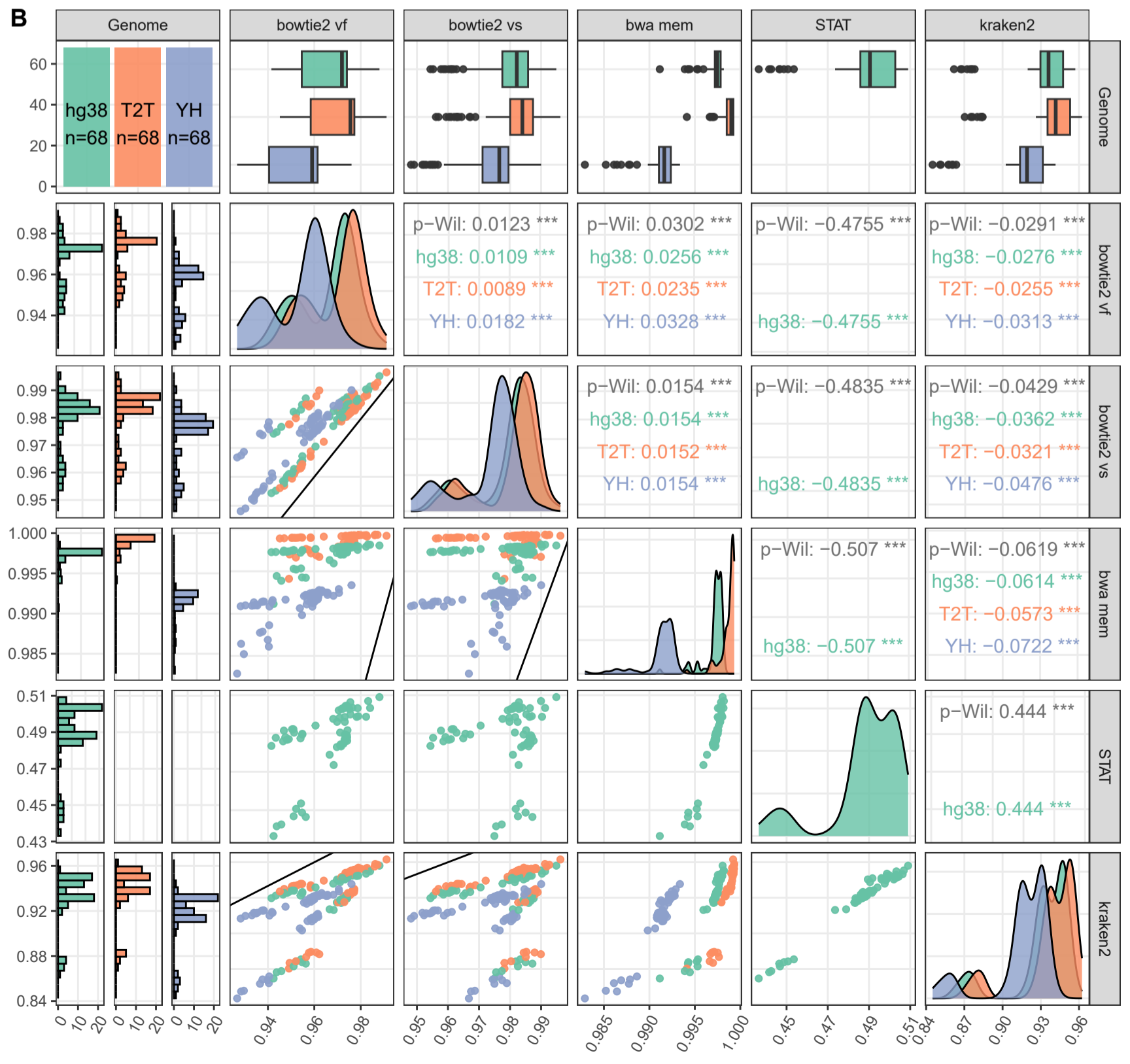

Supplement: Fig. S2 — Comparison of sensitivity. [file msystems.00840-25-s0002.pdf]

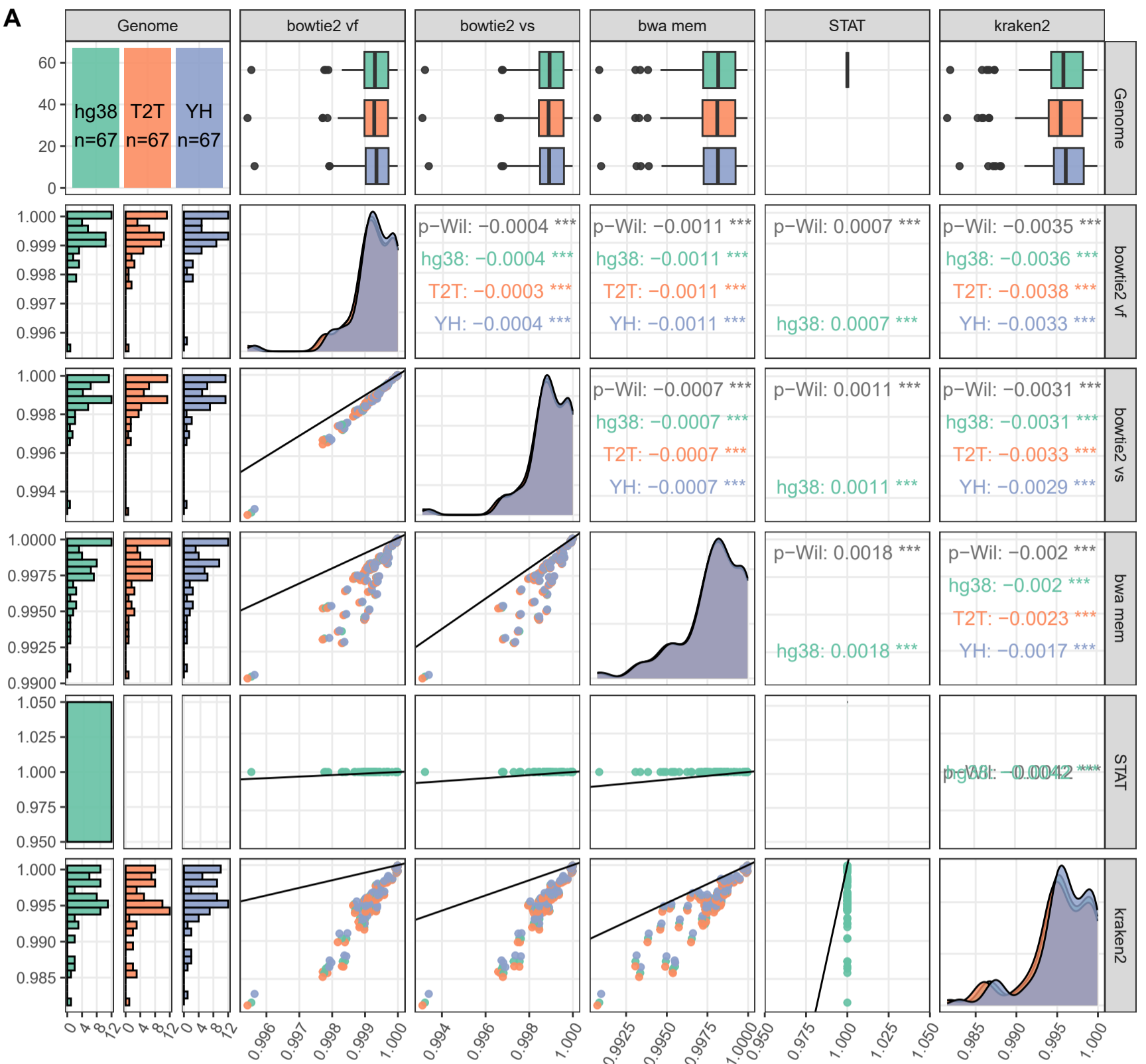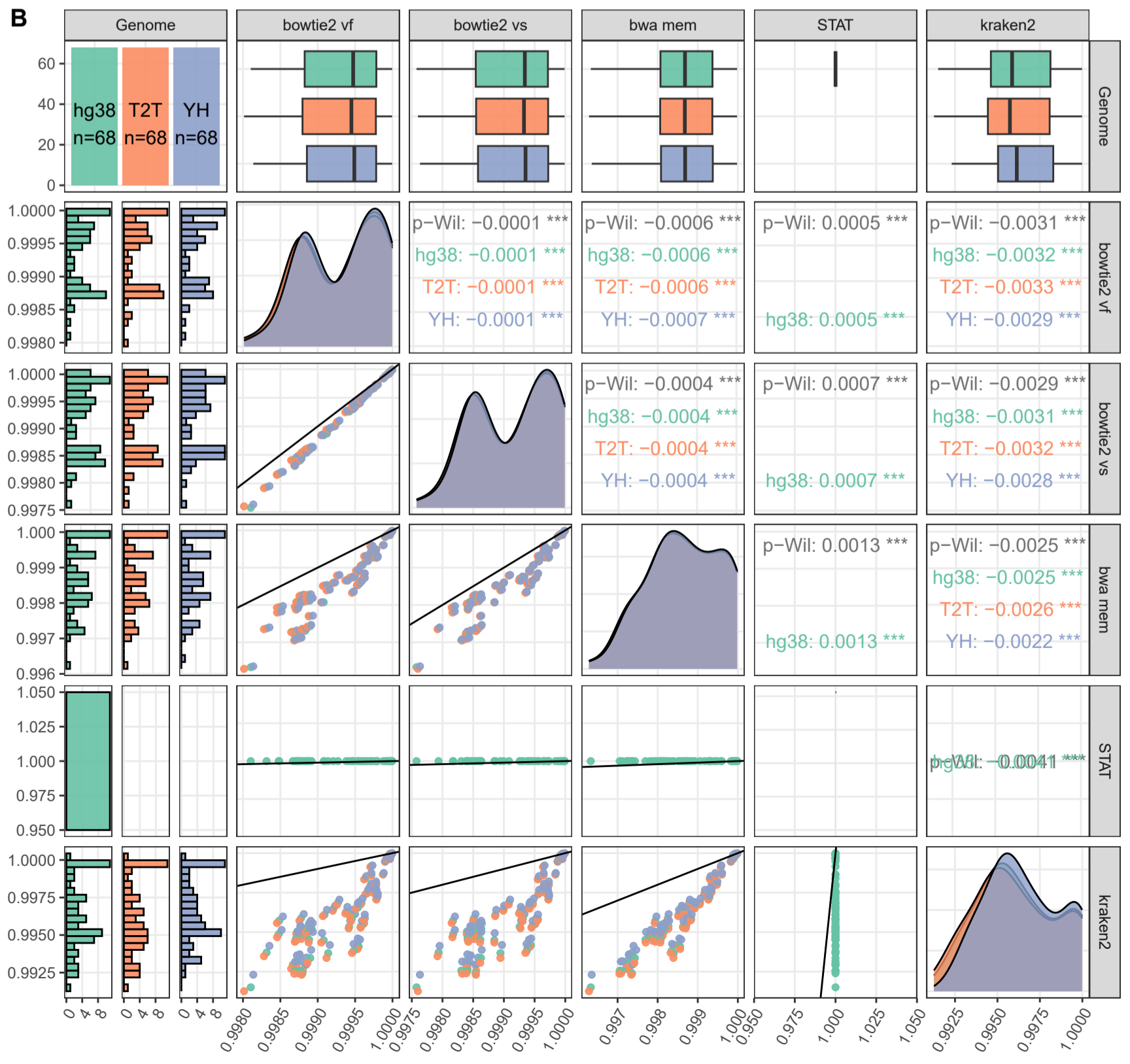

Supplement: Fig. S3 — Comparison of specificity. [file msystems.00840-25-s0003.pdf]

ILMN

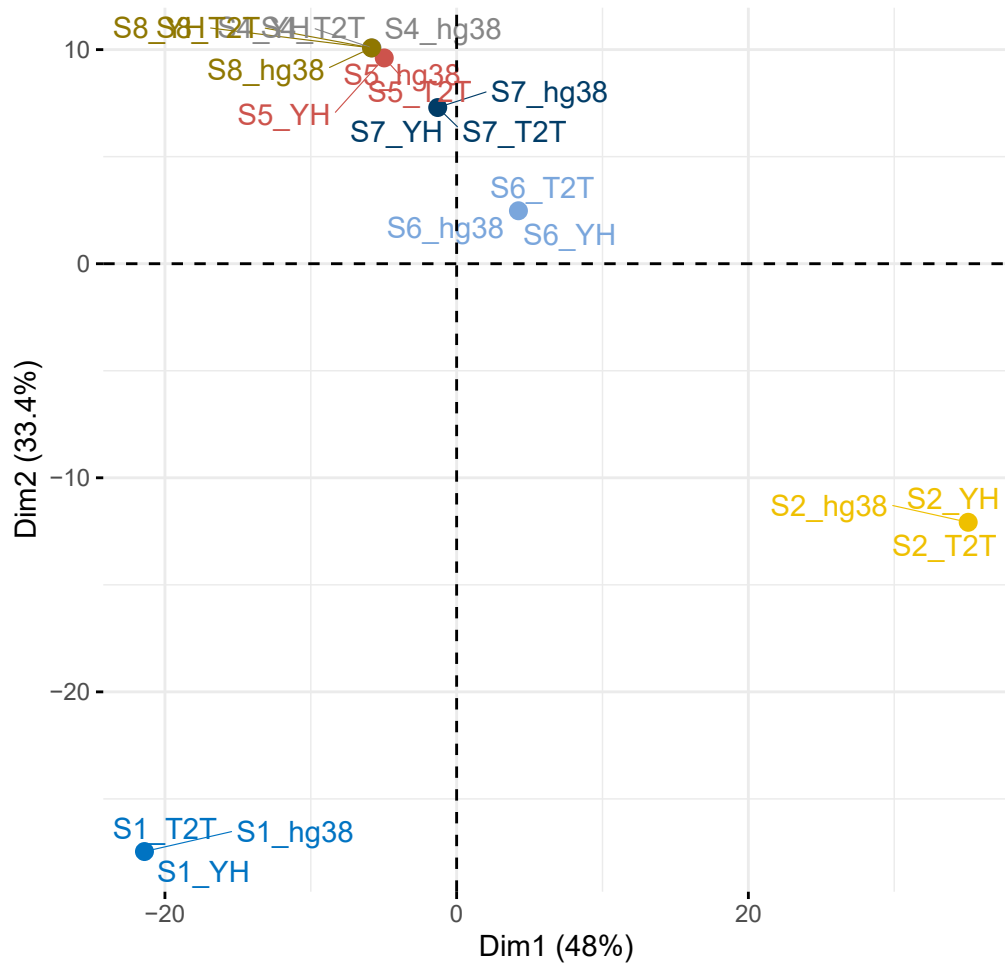

MGI

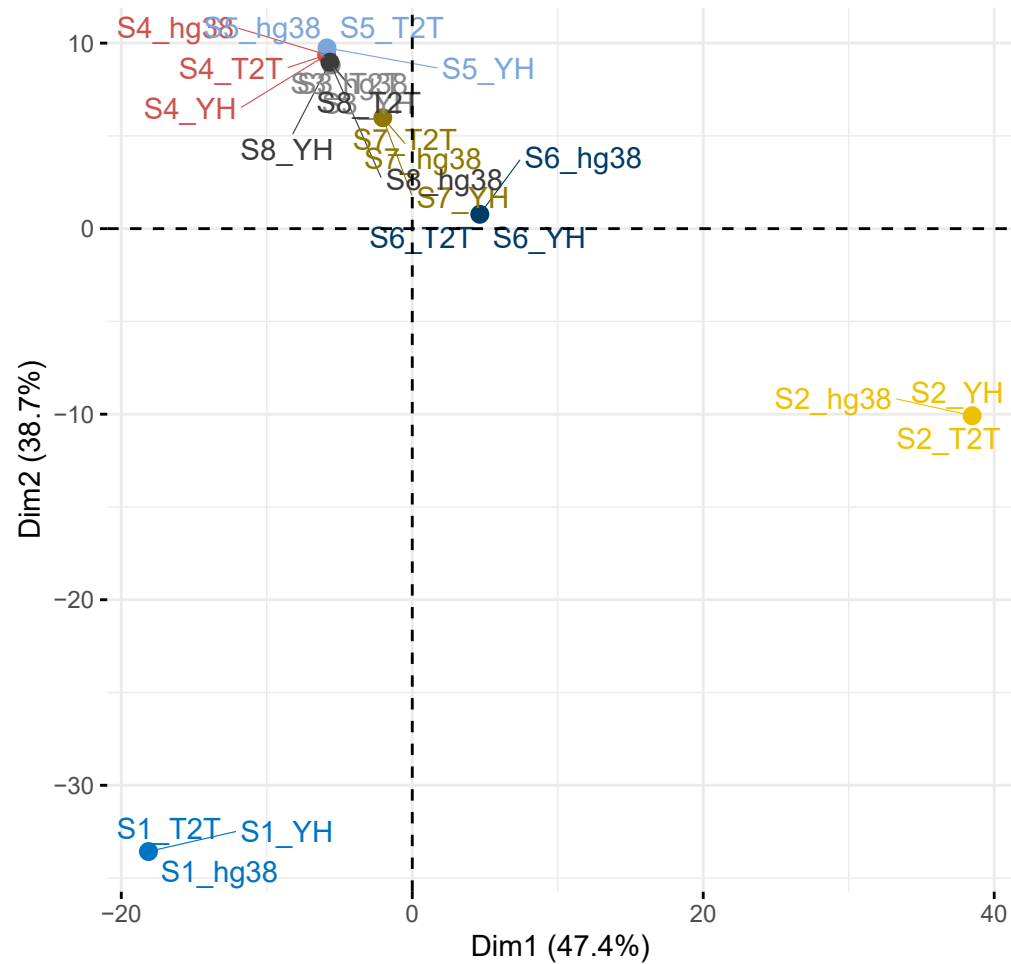

Sample

|        |        |        |        |
|--------|--------|--------|--------|
| ● S1_D | ● S3_D | ● S5_D | ● S7_D |
| ● S2_D | ● S4_D | ● S6_D | ● S8_D |

Supplement: Fig. S4 — Principal component analysis of commensal organisms in the clinical samples. [file msystems.00840-25-s0004.pdf]

Chr9: 48424795-76854863

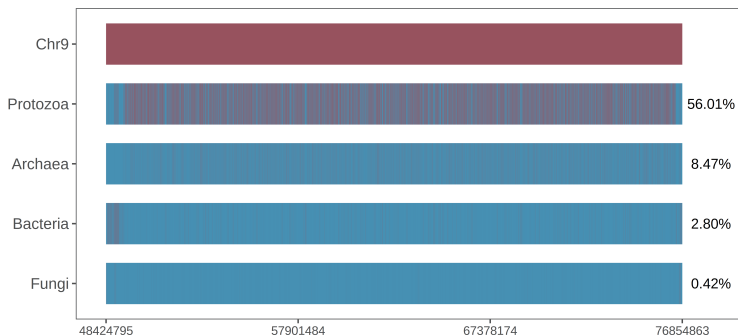

Chr16: 41404411-52028806

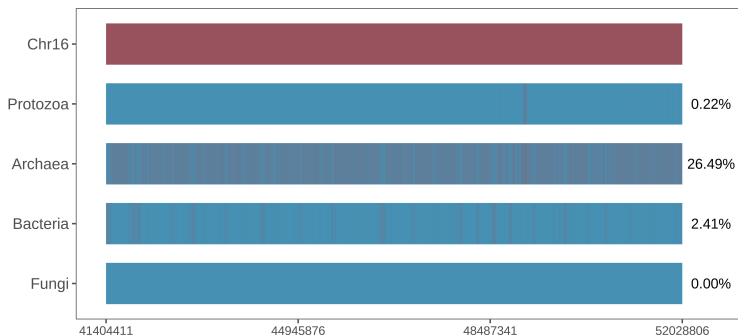

Supplement: Fig. S5 — The contaminative patterns of the T2T exclusive sequences for the database of the 4 taxa. [file msystems.00840-25-s0005.pdf]
