# Supplementary material for: Use of the CHM13-T2T genome improves metagenomic analysis by minimizing host DNA contamination
Source: mSystems. 2025 Sep 10;10(10):e00840-25. doi: 10.1128/msystems.00840-25 (PMC12542756; doi:10.1128/msystems.00840-25)
Supplement: Legends — for supplemental tables and figures. [file msystems.00840-25-s0006.docx]

**Table S1 Description of mock samples.**

**Table S2 Description of clinical samples.**

**Table S3 Comparison results of different dehosting strategies after ILMN platform sequencing.**

**Table S4 Comparison results of different dehosting strategies after MGI platform sequencing.**

**Table S5 Number and total length of human-originated contaminant dectected in each microbial taxa via CHM13-T2T and hg38.**

**Table S6 Information of severely contaminated genomes.**

**Fig. S1 Workflow of collating host reads removal results of all methods.** Classification results from all combination of genomes and algorithms are collated together to establish the "gold standard".

**Fig. S2 Comparison of sensitivity.** Sensitivity comparison across all reference genomes and relevant algorithms. The first column represents the sensitivity of each sample calculated with different algrithms vesus different genomes: hg38 (green), CHM13-T2T (orange, abbreviated as “T2T”), and YH (purple). The first row represents the sum of sensitivity values for each calculation. The diagonal represents the density distribution of the boxplot. Plots in the upper triangle: paired Wilcoxon (p-Wil) comparisons between each combination of reference genomes and algorithms. Numbers are median of differences between the pair, asterisks show the significance of adjusted p-values (no label: insignificant, *: <0.05, **: <0.01, ***: <0.001), and numbers in colors show the comparison for each genome. Plots in the lower triangle: scatterplot of sensitivity. Identity line (y=x) is shown in black, therefore points (each representing a sample) above it have higher sensitivity in y-axis than x-axis and vice versa. Abbreviations are vf: very fast, vs: very sensitive. Results of different sequencers were arranged in **A** for ILMN and **B** for MGI.

**Fig. S3 Comparison of specificity.** Specificity comparison across all reference genomes and relevant algorithms. The first column represents the specificity of each sample calculated with different algrithms vesus different genomes: hg38 (green), CHM13-T2T (orange, abbreviated as “T2T”), and YH (purple). The first row represents the sum of specificity values for each calculation. The diagonal represents the density distribution of the boxplot. Plots in the upper triangle: paired Wilcoxon (p-Wil) comparisons between each combination of reference genomes and algorithms. Numbers are median of differences between the pair, asterisks show the significance of adjusted p-values (no label: insignificant, *: <0.05, **: <0.01, ***: <0.001), and numbers in colors show the comparison for each genome. Plots in the lower triangle: scatterplot of specificity. Identity line (y=x) is shown in black, therefore points (each representing a sample) above it have higher specificity in y-axis than x-axis and vice versa. Abbreviations are vf: very fast, vs: very sensitive. Results of different sequencers were arranged in **A** for ILMN and **B** for MGI.

**Fig. S4 Principle Component Analysis of Commensal Organisms in the Clinical Samples.** The analysis of the composition of commensal organisms is based on classification by kraken2 after removing host reads with correspondent human reference genomes. Each point is marked by the combination of sample ID and the reference genome used. A good separation of samples is evident, but in general the results of all three genomes are identical and overlap with each other. “CHM13-T2T” was abbreviated as “T2T”.

**Fig. S5 The contaminative patterns of the CHM13-T2T exclusive sequences for the database of the 4 taxa.** Red segments represent contaminant regions.
